# Analgesic and anti-inflammatory activity of *Argyreia speciosa* root

**DOI:** 10.4103/0253-7613.56066

**Published:** 2009-08

**Authors:** R.S. Bachhav, V.S. Gulecha, C.D. Upasani

**Affiliations:** Department of Pharmacology, S.N.J.B'S S.S.D.J. College of Pharmacy Chandwad, Dist. Nashik - 423 101, Maharashtra, India; 1 NGSPM's College of Pharmacy, Brahma Valley Educational Campus At/p Anjaneri Tal- Trimbak, Nashik, India

**Keywords:** *Argyreia speciosa*, anti-inflammatory, analgesic, methanolic extracts

## Abstract

**Objective:**

To study analgesic and anti-inflammatory activities of a methanolic extract (ME) of *Argyreia speciosa* (AS) root powder.

**Materials and Methods:**

The study was carried out using male albino mice (20-25 gm) and male wistar rats (100-150gm). The ME was prepared using soxhlet extraction process. The effect of ME of *A. speciosa* was investigated for analgesic activity using acetic acid-induced abdominal constriction, tail immersion method and hot plate method. The anti-inflammatory activity of ME of AS roots was studied using carrageenan-induced rat paw edema.

**Result:**

The ME of *A. speciosa* root was used in pain and inflammation models. The analgesic activity of AS at the dose of (30,100, and 300 mg/kg p.o) showed significant (*P*<0.01) decrease in acetic acid-induced writhing, whereas ME of *A. speciosa* at the dose of (100, 300 mg/kg p.o) showed significant (*P*<0.01) increase in latency to tail flick in tail immersion method and elevated mean basal reaction time in hot plate method. The ME of the *A. speciosa* at doses (30, 100, and 300mg/kg) showed significant (*P* < 0.01) inhibition of carrageenan induced hind paw edema in rats.

**Conclusion:**

The ME of *A. speciosa* showed significant analgesic and anti-inflammatory activity in mice and rat.

## Introduction

*Argyreia speciosa* Burm. F. (Convolvulaceae), commonly known as *Vridha daraka* is a rasayana herb used in many Ayurvedic preparations in the Indian system of medicine. Roots of *A. speciosa* are used in Ayurveda as an aphrodisiac, rejuvenator, intellect promoting brain tonic, nervine tonic for liver disorders and as a tonic for general debility.[1–3] Previous investigations on *A. speciosa* roots have revealed the presence of aryl esters,[4] coumarin glucoside,[5] p-hydroxycinnamate and scopoletin[6] as important phytoconstituents. In Hindu medicine, the root is regarded as an alternative tonic and useful in rheumatic affection and disease of the central nervous system. The plant has been reported to possess antimicrobial,[6] anti-inflammatory, wound healing,[7] immunomodulatory[8] and nootropic[9] activities. This study was undertaken to evaluate the analgesic and anti-inflammatory properties of methanolic extract of *A. speciosa* in mice and rats at different doses.

## Materials and Methods

### Preparation of methanolic extract

The roots were obtained from the garden of Ayurvedic Mahavidyalay, Panchvati Ganeshwadi, Nashik 422003. About 500g of the powder was subjected to soxhlet extraction for 12 hours using 5.0 liters of methanol as a solvent. The extract was made free from solvent by keeping it on a water bath at 50-60°C and gave a yield of 20%.

### Phytochemical test

The freshly prepared ME of *A. speciosa* was subjected to standard phytochemical screening tests for various constituents.

### Animals

Albino mice (20-25 gm) and Wistar rats (100-150 gm) were obtained from the National Toxicological Centre, Sinhgad road, Pune. Animals were housed in groups of five at an ambient temperature of 25±1°C with free access to food and water. Animals were deprived of food but not water four hours before the experiment. For screening of analgesic and anti-inflammatory activity, mice and rats were divided into five different groups. The first group served as a control group. The second group was used as reference standard. Three groups received ME of *A. speciosa* at three different doses (30,100,300 mg/kg p.o.). The study protocol was approved by Institutional Animal Ethics Committee.

### Drugs

Aspirin (Research Lab, Mumbai), pentazocine (Fortwin, Ranbaxy) and carrageenan (Sigma, Mumbai) were used in the study. The ME of *A. speciosa* (30, 100, and 300 mg/kg), carrageenan and aspirin were prepared in two per cent gum acacia suspension before oral administration (p.o). Pentazocine was mixed in saline before intra peritoneal administration (i.p). Two per cent gum acacia suspension prepared in distilled water was used in the control group.

### Acetic acid-induced writhing

In this method, mice were divided in five groups of five each. The animals were pretreated with drugs 45 minutes before induction of writhing. The animals received the standard drug aspirin (20 mg/kg, p.o.) which served as reference standard. Analgesic activity of ME of *A. speciosa* (30,100, and 300 mg/kg, p.o.) was assessed by counting the number of writhes induced by 0.6% acetic acid (10 ml/kg i.p.).[10,11] The number of writhes per animal was counted for the next 20 minutes. Percentage protection against abdominal constriction was taken as an index of analgesia.

It was calculated as: $\frac{\text{Number of writhing in control group - Number of writhing in treated group}}{\text{Number of writhing in control group}}\text{ X 100}$

### Tail immersion method

In this method, mice were divided in five groups of five each. The animals were pretreated with drugs 60 minutes before tail immersion. The animals received the standard drug pentazocine (17.5 mg/kg, i.p.) which served as reference standard. The distal 2-3 cm portion of mouse-tail was immersed in hot water maintained at 55±1°C.[11] The time taken by the mouse to withdraw the tail from hot water was noted as reaction time. The cut off time was considered 10-12sec.

### Hot plate method

In this method, mice were divided in five groups of five in each group. The animals were pretreated with drugs 60 minutes before experimentation. The animals received the standard drug aspirin (20 mg/kg, p.o.) served as reference standard. They were placed on a hot plate maintained at a temperature of 55±0.5°C.[11] The latency to flick the hind paw or lick or jump from the hot plate was the reaction time. The reaction time was noted at 0, 15, 30, 45, 60, 90, and 120 min. The cut off time was considered as 15 seconds.

## Anti-inflammatory Activity

### Carrageenan-induced rat paw edema

In this method, rats were divided in five groups of five each. The animals were pretreated with drugs 60 minutes before injection of carrageenan (0.1 ml of 1%). Carrageenan was injected into the sub plantar tissue of left hind paw of each rat. Swellings of carrageenan-injected foot were measured at zero, one, two, three hours using Plethysmometer (UGO Basile, Italy).[12,13] The right hind paw was injected with 0.1 ml of vehicle. The animals received the standard drug aspirin (20 mg/kg, p.o.) which served as reference standard.

### Statistical analysis

Results were expressed as mean ± SEM. Statistical analysis was performed using one-way analysis of variance (ANOVA) followed by Dunnett's test. *P* < 0.05 was considered statistically significant.

## Results

### Phytochemical test

The phytochemical screening revealed the presence of alkaloids, resins, carbohydrate, flavonoids, triterpenes, starch, and tannins in ME of *A. speciosa*.

### Acetic acid-induced abdominal constriction method

**Figure 1:**
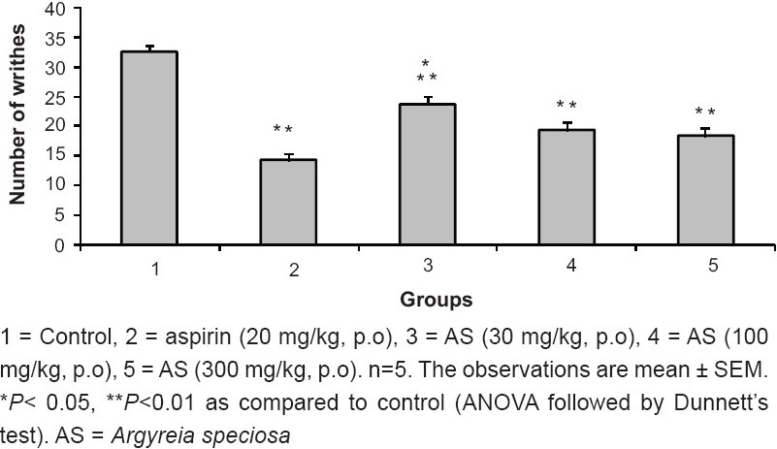
Effect of ME of *A. speciosa* (30, 100, 300, mg/kg) on acetic acid-induced abdominal constriction in mice

The ME of *A. speciosa* (30, 100 and 300 mg/kg, p.o.) significantly (*P* < 0.01) reduced the number of abdominal constrictions induced by acetic acid compared to control group. Maximum inhibition of writhing response by ME of *A. speciosa* (300 mg/kg) was 43.55%, which was comparable to aspirin (20 mg/kg) [Figure 1].

### Tail immersion method

**Figure 2:**
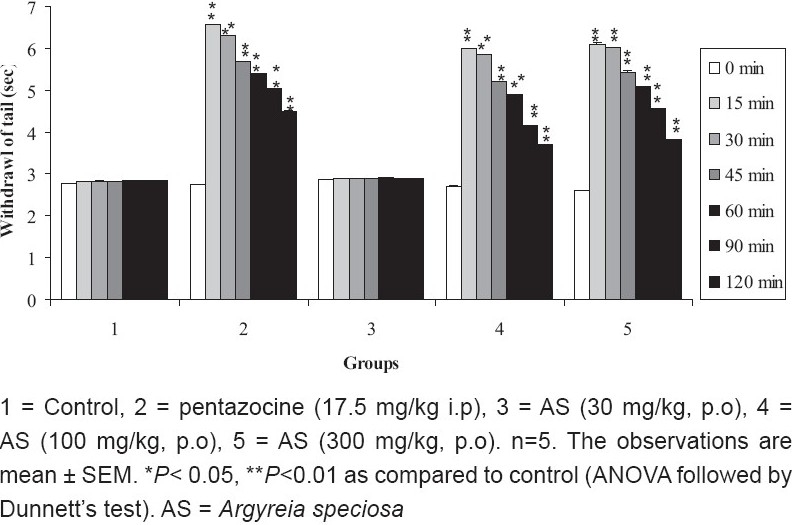
Effect of ME of *A. speciosa* (30, 100, and 300 mg/kg) on mean reaction time by tail immersion method in mice

The ME of *A. speciosa* (30 mg/kg, p.o.) did not show significant increase in latency to flick tail compared to control group (*P* > 0.05). The ME of *A. speciosa* at doses (100 and 300 mg/kg, p.o.) showed significant (*P* < 0.01) increase in latency to flick tail compared to control group. The highest nociception inhibition of stimulus by ME of *A. speciosa* (300 mg/kg) was observed at 15 minutes [Figure 2].

### Hot plate method

**Figure 3:**
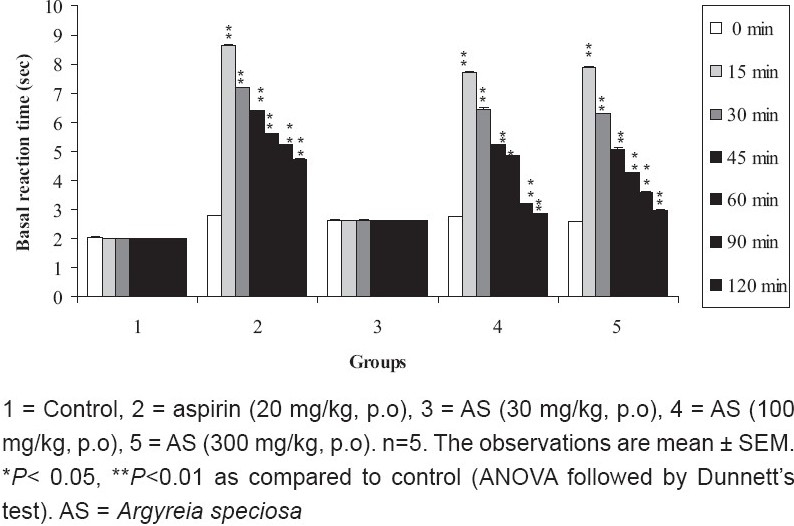
Effect of ME of *A. speciosa* (30, 100, and 300 mg/kg) on mean reaction time by hot plate method in mice

The ME of *A. speciosa* (30mg/kg, p.o.) did not show significant (*P* > 0.05) increase in the mean basal reaction time in hot plate method compared to control. The ME of *A. speciosa* at doses (100 and 300 mg/kg, p.o.) showed significant (P < 0.01) increase in the mean basal reaction time. The highest nociception inhibition of stimulus exhibited by ME of *A. speciosa* (300 mg/kg) was observed at 15 minutes [Figure 3].

Carrageenan-induced Rat Paw Edema

**Figure 4:**
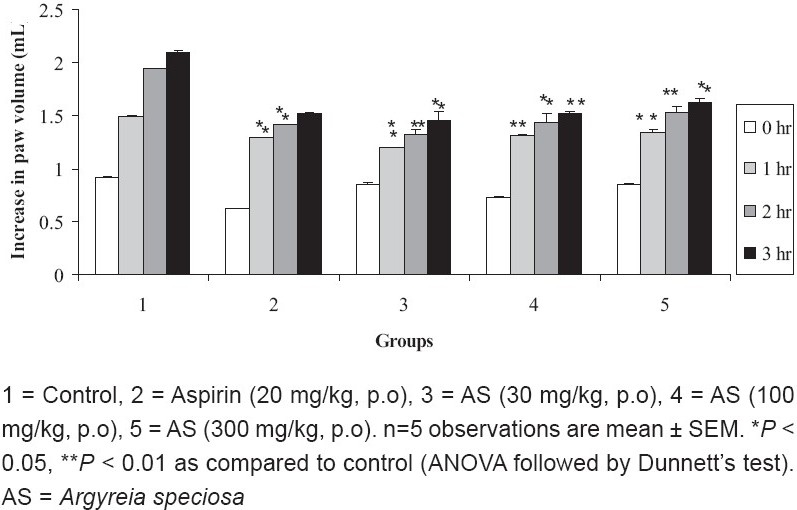
Effect of ME of *A. speciosa* (30, 100, and 300 mg/kg) on carrageenan-induced rat paw edema

The ME of *A. speciosa* (30, 100, and 300 mg/kg, p.o.) significantly (P < 0.01) inhibited carrageenan induced rat paw edema. Maximum inhibition of paw edema was observed in ME of *A. speciosa* (100 mg/kg) at four hours when compared to the control group. Aspirin inhibited carrageenan induced rat paw edema by 40.93% [Figure 4].

## Discussion

The preliminary phytochemical screening of ME of *A. speciosa* show the presence of alkaloids, resins, carbohydrate, flavonoids, triterpenes, starch, and tannins.[14] Thus, the activity of *A. speciosa* could be due to flavonoids and triterpene components.[15] The ME of *A. speciosa* did not show any toxicity and behavioral changes in mice up to 3000 mg/kg hence doses of (30, 100, and 300 mg/kg, p.o.) were selected for the present study.[16] The analgesic and anti-inflammatory effect of ME of *A. speciosa* in various models of pain and inflammation were found to be analogous. The stimulus may be thermal (tail flick, tail immersion, and hot plate tests), mechanical (tail or paw pressure tests), electrical (stimulation of paw, tail or dental pulp) or chemical (‘writhing’ and formalin tests).[17] Acetic acid-induced abdominal constriction is a sensitive method for screening peripheral analgesic effect of compounds. It causes an increase in concentration of PGE2 and PGF2α in the peritoneal fluid.[18,19]

The hot plate method and tail immersion method have been found to be suitable for evaluation of centrally acting analgesics.[20] The nociceptors seem to be sensitized by sensory nerves. The involvement of endogenous substances such as PGs may be minimized in this model. In centrally acting analgesic methods, the drug in100 mg/kg and 300 mg/kg doses was found to be significantly effective. The 30 mg/kg dose was found to be ineffective in this analgesic model for evaluating centrally acting drugs. However, all the three doses were found to be effective in acetic acid-induced abdominal constriction method, that is used to evaluate peripherally acting analgesics In the acetic acid-induced abdominal constriction method, pain is generated indirectly via endogenous mediators like prostaglandin, which stimulates peripheral nociceptive neurons. These neuronal fibers are sensitive to both narcotics and non steroidal anti-inflammatory drugs.[19] The ME of *A. speciosa* inhibited the acetic acid-induced pain with potency compared to the aspirin. The standard drug aspirin which inhibit the peripheral pain induced by direct action of acetic acid in the abdomen by inhibiting the prostaglandin secretion.

Carrageenan-induced rat paw edema has been a popular inflammatory model to investigate anti-inflammatory effect of compounds.[21] It has a biphasic effect. The first phase is due to release of histamine and serotonin (5-HT) (0-2 hr), plateau phase is maintained by a kinin like substance (3hr) and second accelerating phase of swelling is attributed to PG release (>4hr).[22] In our study, ME of *A. speciosa* (30, 100, and 300 mg/kg, p.o.) significantly reduced edema induced by carrageenan in all three phases. Hence it can be concluded that ME of *A. speciosa* possesses analgesic and anti-inflammatory properties that may be mediated by the presence of triterpene and flavonoid components.
